# Method of Shortening Some Tedious Labors

**Published:** 1861-01

**Authors:** 


*Method of Shortening some Tedious Labors.*—M. de Laffore read to the French Academy of Medicine, a memoir on tedious natural parturition, and an innocuous mode of shortening the duration of labor. The author seeks to demonstrate that one of the chief obstacles to parturition is the resistance which the symphysis presents to the passage of the fœtus. Hence, for the purpose of accelerating labor, it would suffice, in most cases, to apply the forefinger upon the anterior part of the cervix, so as to keep the presenting part from coming into contact with the symphysis.

British obstetricians have as long ago as 1837 observed this same difficulty during labor. Drs. Hamilton, Burns and Brien have advised that the anterior lip, which is often constricted and swollen between the head and the symphysis, should be pushed up by the finger in the interval of the pains, and there maintained until the returning contraction of the uterus drives the head below it. Drs. Collins and Murphy objected to the practice, as likely to produce inflammation of the cervix. Dr. Murphy recommends instead the pressure upon the head which has since been described by Dr. de Laffore.—(*Championniere's Journal*, *October and November.*)

